# Growth performance, oxidative stress and immune status of newly weaned pigs fed peroxidized lipids with or without supplemental vitamin E or polyphenols

**DOI:** 10.1186/s40104-020-0431-9

**Published:** 2020-03-05

**Authors:** Y. V. Silva-Guillen, C. Arellano, R. D. Boyd, G. Martinez, E. van Heugten

**Affiliations:** 1 Department of Animal Science, North Carolina State University, Raleigh, NC 27695 USA; 2 Department of Statistics, North Carolina State University, Raleigh, NC 27695 USA; 3 The Hanor Company Inc, Franklin, KY 42134 USA

**Keywords:** Antioxidants, Immune status, Lipid peroxidation, Oxidative stress, Piglets, Polyphenols, Vitamin E

## Abstract

**Background:**

This study evaluated the use of dietary vitamin E and polyphenols on growth, immune and oxidative status of weaned pigs fed peroxidized lipids. A total of 192 piglets (21 days of age and body weight of 6.62 ± 1.04 kg) were assigned within sex and weight blocks to a 2 × 3 factorial arrangement using 48 pens with 4 pigs per pen. Dietary treatments consisted of lipid peroxidation (6% edible soybean oil or 6% peroxidized soybean oil), and antioxidant supplementation (control diet containing 33 IU/kg *DL*-*α*-tocopheryl-acetate; control with 200 IU/kg additional dl-α-tocopheryl-acetate; or control with 400 mg/kg polyphenols). Pigs were fed in 2 phases for 14 and 21 days, respectively.

**Results:**

Peroxidation of oil for 12 days at 80 °C with exposure to 50 L/min of air substantially increased peroxide values, anisidine value, hexanal, and 2,4-decadienal concentrations. Feeding peroxidized lipids decreased (*P* < 0.001) body weight (23.16 vs. 18.74 kg), daily gain (473 vs. 346 g/d), daily feed intake (658 vs. 535 g/d) and gain:feed ratio (719 vs. 647 g/kg). Lipid peroxidation decreased serum vitamin E (*P* < 0.001) and this decrease was larger on day 35 (1.82 vs. 0.81 mg/kg) than day 14 (1.95 vs. 1.38 mg/kg). Supplemental vitamin E, but not polyphenols, increased (*P* ≤ 0.002) serum vitamin E by 84% and 22% for control and peroxidized diets, respectively (interaction, *P* = 0.001). Serum malondialdehyde decreased (*P* < 0.001) with peroxidation on day 14, but not day 35 and protein carbonyl increased (*P* < 0.001) with peroxidation on day 35, but not day 14. Serum 8-hydroxydeoxyguanosine was not affected (*P* > 0.05). Total antioxidant capacity decreased with peroxidation (*P* < 0.001) and increased with vitamin E (*P* = 0.065) and polyphenols (*P* = 0.046) for the control oil diet only. Serum cytokine concentrations increased with feeding peroxidized lipids on day 35, but were not affected by antioxidant supplementation (*P* > 0.05).

**Conclusion:**

Feeding peroxidized lipids negatively impacted growth performance and antioxidant capacity of nursery pigs. Supplementation of vitamin E and polyphenols improved total antioxidant capacity, especially in pigs fed control diets, but did not restore growth performance.

## Background

Swine diets often include fats or oils to increase the energy density of the diet. Fats and oils are very dense energy sources with a low cost per unit of energy [1]. However, supplemental lipids are prone to peroxidation due to oxygen attack on unsaturated fatty acids when exposed to high temperatures [2, 3]. Unsaturated lipids are more susceptible to peroxidation than saturated fatty acids [2]. Thus, vegetable oils are more prone to peroxidation than more saturated fats, such as choice white grease and tallow [3]. High amounts of vegetable oils are used in the preparation of foods for human consumption. This cooking process at high temperatures produces high amounts of peroxidized oils [4]. Commonly, spent cooking oils are recycled into lipid sources for use in animal feed [5].

Studies evaluating the impact of peroxidized oils in livestock have shown variable results, including no effects on growth performance of rabbits [6], a reduction in feed intake in sheep [7], pigs, and broilers [8], a reduction in growth of pigs [9–13], broilers [8, 14, 15], and rats [16], increased morbidity and mortality in pigs [17], reduced oxidative stability of pork [18], no oxidative effects of sheep meat [19], alteration of the nutritional quality of chicken and rabbit meat [20], and compromised oxidative status in animals [4, 14–16, 21] causing degradation of cellular components, including protein, lipids, and DNA [15, 21].

Natural antioxidants in the body play an important role to delay or prevent oxidation and blocking and capturing formed radicals in cells [22]. Vitamin E is a fat-soluble vitamin and natural antioxidant [23, 24]. Likewise, plant-based polyphenols are antioxidants with high potential against oxidation [25]. Several studies reported positive effects of vitamin E or plant polyphenols on meat oxidation in broilers [26, 27], meat quality in finishing pigs [9], and antioxidant activity in piglets [28, 29]. Moreover, the supplementation of vitamin E plus synthetic antioxidant blends or synthetic antioxidants to diets containing peroxidized vegetable oil for weaned piglets and finishing pigs improved growth and reduced lipid and protein oxidation [9, 30].

We hypothesized that supplemental antioxidants could alleviate the negative effects of peroxidized lipids by improving the antioxidant status of pigs and reducing oxidative stress. The objective of the current study was to investigate the potential antioxidant effects of vitamin E or plant polyphenols when supplemented to diets containing peroxidized oil on growth performance, immune and oxidative status, and antioxidant function of weaned pigs.

## Material and methods

### Animals, treatments and experimental design

This study was conducted at the Swine Educational Unit (Raleigh, NC). One-hundred and ninety-two 3-week old crossbred pigs (96 gilts and 96 barrows) with an initial body weight of 6.62 ± 1.04 kg were used. At weaning, piglets were blocked by initial body weight and sex and randomly assigned within blocks to a 2 × 3 factorial arrangement using an experimental allotment program [31]. Factors consisted of: 1) dietary inclusion of 2 types of oils [6% refined, bleached, and deodorized soybean oil (control oil) or 6% of the same soybean oil that had been peroxidized (peroxidized oil)] and 2) inclusion of 3 antioxidant treatments consisting of a control diet (33 IU/kg of *DL*-*α*-tocopheryl acetate; CON), the control diet with an additional 200 IU/kg of *DL*-*α*-tocopheryl acetate (Rovimix®, DSM Nutritional Products, Parsippany, NJ; VITE) or the control + 400 mg/kg of a blend of plant-based polyphenols (Promote® AOX™ 50, Cargill, Inc., Wayzata, MN; POL), based on the manufacturers’ recommendation. The vitamin E concentration in the control diet was representative of current industry practices [32] and supplementation with 200 IU/kg was based on previous research [33].

The peroxidized oil was created using 163 kg of refined, bleached, and deodorized soybean oil (Kirkland, Washington, WA), equally divided into two metal barrels. A heater was placed into each barrel and was set to maintain a constant temperature of 80 °C for 12 days. In addition, a polyvinyl chloride pipe with 1 mm holes was submerged in each barrel to continuously bubble air through the oil at a constant flow rate of 50 L/min for 12 days. The dietary treatment feeds were manufactured at the North Carolina State University Feed Mill Educational Unit (Raleigh, NC). Diets were based primarily on corn-soybean meal and were formulated to meet or exceed all nutrient requirements for piglets as suggested by the NRC (2012) [1]. At the time of mixing, 0.1% of liquid antioxidant (Rendox®, Kemin Industries, Inc., Des Moines, IA) containing tertiary butyl hydroquinone (TBHQ) was added to the control oil and the peroxidized oil prior to mixing of feed to avoid further peroxidation. A basal mix containing all ingredients, except the oil sources and supplements was created and equally divided into six batches. Final treatment diets were then created by mixing control oil or peroxidized oil with the basal mix and within these, no supplement, vitamin E, or polyphenols were added at the appropriate concentrations. Diets were fed in 2 phases throughout the 5-week nursery period, with phase 1 being fed immediately after weaning for 14 days and phase 2 being fed the next 21 days (Table 1). All piglets were provided ad libitum access to feed and drinking water.

**Table 1:** Composition of the experimental Phase 1 and 2 diets. As fed basis^a^

Item	Phase 1	Phase 2
Ingredient, %		
Corn, yellow dent	36.35	55.34
Soybean meal, 47.5% CP	21.00	34.67
Whey permeate	20.00	–
Poultry byproduct meal	6.76	–
Fish meal, menhaden	4.00	–
Plasma, spray-dried	4.00	–
Soybean oil^b^	6.00	6.00
Monocalcium phosphate, 21% P	0.35	1.53
Limestone	0.24	1.16
Zinc oxide, 72% Zn	0.26	–
Copper sulfate, 25.2% Cu	0.07	0.07
*L*-lysine HCl	0.27	0.36
*DL*-methionine	0.20	0.18
*L*-threonine	0.13	0.14
*L*-tryptophan	0.01	–
Salt	0.15	0.35
Mineral premix^c^	0.15	0.15
Vitamin premix^d^	0.05	0.05
Calculated Composition		
ME, kcal/kg	3609	3556
Crude protein, %	24.2	21.7
Total lysine, %	1.70	1.45
Ca, %	0.80	0.80
P, %	0.76	0.72
Standardized ileal digestible amino acids, %		
Lys	1.50	1.30
Thr	0.93	0.81
Met	0.53	0.47
Met+Cys	0.87	0.75
Trp	0.27	0.23

^a^Diets were formulated to meet or exceed NRC (2012) recommendations. Phase 1 diets were fed from day 0 to 14 and Phase 2 diets were fed from day 14 to day 35. Experimental diets were created from a common basal mix to which the appropriate oil source was added, and by replacing corn in the control diet with vitamin E (Rovimix®, DSM Nutritional Products, Parsippany, NJ) to supply 200 IU/kg of *DL*-*α*-tocopheryl acetate, or 400 mg/kg of a blend of plant-based polyphenols (Promote® AOX^t^™ 50, Cargill, Inc., Wayzata, MN)

^b^ Control or peroxidized soybean oil. Peroxidation was created by heating control oil at 80 °C while bubbling air through oil at the rate of 50 L/min for 12 d. Control and peroxidized soybean oil were stabilized with 0.1% liquid antioxidant containing tertiary butyl hydroquinone after peroxidation was completed. Analyzed dietary crude fat concentrations were 7.74% and 6.96% for Phase 1 and 5.34% and 4.01% for Phase 2 for control and peroxidized oil treatments, respectively

^c^Supplied per kg of complete diet: 33 mg of Mn, 110 mg of Zn, 110 mg of Fe, 17 mg of Cu, 0.30 mg of I and 0.30 mg of Se

^d^Supplied per kg of complete diet: 8,270 IU of vitamin A, 1,650 IU of vitamin D_3_, 33 IU of vitamin E, 0.04 mg of vitamin B_12_ as *D*-calcium pantothenate, 3.3 mg of vitamin K as menadione sodium bisulfite complex, 7.7 mg of riboflavin, 25.0 mg of d-pantothenic acid, 44.1 mg of niacin, 0.11 mg of biotin

Pigs and treatments were randomly allotted within body weight and sex blocks into 2 identical temperature-controlled rooms (Aerotech®, Aero Speed 1.2, Pittsburgh, PA) containing 24 pens each (48 pens total with 8 pen replicates per treatment). Pigs were housed 4 pigs per pen (2 gilts and 2 barrows). The size of each pen was 0.91 m × 1.52 m and each pen contained a stainless-steel single-sided feeder with 2 feeding spaces (Staco, Inc., Schaeffer town, PA) and 2 stainless steel water nipples for weaned piglets (Hog Slat, Newton Grove, NC). Environmental temperatures, ventilation rate, and pigs were checked every morning. Temperatures were set at 32 °C for the first week and then reduced by 2 °C throughout each week until the temperature reached 24 °C.

### Sampling and measurements

Individual pig body weight (BW) was measured on days 0, 7, 14, 21, 28 and 35 to calculate average daily gain (ADG) for each pen. Average daily feed intake (ADFI) was measured from the difference between the sum of feed additions and feed remaining at the end of the week or phase and divided by 7 days or days in the phase. Feed efficiency (G:F) was calculated by dividing ADG by ADFI weekly and for each phase.

Blood samples from one randomly selected pig per pen were collected by venipuncture using 20-gauge × 3.8 cm multiple use drawing needles (Vacuette®, Greiner bio-one, Kremsmunster, Austria) on day 14 and day 35 (at approximately 09:00). Blood was collected into 10 mL-vacuum tubes for serum (BD Vacutainer®, Franklin Lakes, NJ). Blood was centrifuged at 4,267×*g* for 10 min at 4 °C using a refrigerated centrifuge (Centra GP8R, Thermo IEC, MA) and serum was collected. Serum was aliquoted into 6 tubes of 2 mL capacity (Biotix®, Neptune, 3472.X, Mesa Rim, San Diego, CA) and stored at − 80 °C for further analysis.

### Oil analysis

Representative oil samples from control and peroxidized oil were collected and analyzed by New Jersey Feed Laboratory Inc. (Trenton, NJ) for moisture, insoluble impurities, unsaponifiable matter, free fatty acids, peroxide value (initial, and at 4 and 20 h using the active oxygen method (AOM)), and anisidine value using AOAC [34] and AOCS [35] procedures and by Kemin Industries, Inc. (Des Moines, IA) for oxidative stability index, hexanal, 2,4-decadienal and tertiary butyl hydroquinone.

### Serum oxidative status markers

Malondialdehyde (MDA) was measured using the Oxiselect™ TBARS assay kit (catalog number STA330; Cell BioLabs, Inc., San Diego, CA) and 8-hydroxydeoxyguanosine (8-OHdG) was measured using the Oxiselect™ Oxidative DNA Damage ELISA kit (catalog number STA3208; Cell BioLabs, CA). Absorbance was measured at 532 nm and 450 nm for MDA and 8-OHdG, respectively, on a microplate reader (Bio Tek Instruments®, Synergy HT, Winooski, VT), using a software program (KC4™, Bio Tek Instruments®, Winooski, VT). Results from MDA were expressed in μmol/L. The intra assay CV was 9%. Results for 8-OHdG were expressed in ng/mL. Intra-and inter-assay CV were 4% and 3%, respectively. Protein carbonyl was measured in serum samples using the protein carbonyl colorimetric assay kit (Cayman Chemical®, Ann Arbor, MI). Protein carbonyl was expressed based on protein concentration, which was determined using the bicinchoninic acid (BCA) protein assay kit (Fisher Scientific®, Hampton, NH). Absorbance was measured at 360 nm on a microplate reader (Bio Tek Instruments®, Synergy HT, Winooski, VT), using a software program (KC4™, Bio Tek Instruments®, Winooski, VT). Results were expressed in pmol carbonyl/mg of protein. Intra-and inter-essay CV were 5% and 3%, respectively.

### Serum vitamin E concentrations and total antioxidant capacity

Serum vitamin E concentrations were analyzed in serum samples collected on day 14 and day 35 by the Veterinary Diagnostic Laboratory at Iowa State University (Ames, IA). Samples were analyzed using high performance liquid chromatography. Total antioxidant capacity (TAC) was measured in serum samples using the Oxiselect™ TAC assay kit according to the manufacturers’ protocol (Cell BioLabs, Inc., San Diego, CA, catalog number STA360). Absorbance was measured at 490 nm on a microplate reader (Bio Tek Instruments®, Synergy HT, Winooski, VT), using a software program (KC4™, Bio Tek Instruments®, Winooski, VT). Results were expressed as μmol/L copper reducing equivalents (CRE), which are proportional to the sample’s total antioxidant capacity. Intra-and inter-assay CV were 2.1% and 3.5%, respectively.

### Serum cytokines

Serum samples for both day 14 and day 35 were submitted to Eve Technologies Corporation (Calgary, Canada) for analysis of pro- and anti-inflammatory cytokines using the Luminex xMAP Multi-plex technique. Results of cytokines from interferon- *γ*, IL (interleukin)-1*α*, IL-1 *β*, IL-1ra, IL-2, IL-4, IL-6, IL-8, IL-10, IL-12, IL-18, and tumor necrosis factor- *α* (TNF- *α*) were reported and expressed in pg/mL of serum.

### Statistical analysis

The data for growth performance and serum measurements were analyzed using the Proc MIXED procedure of SAS (v.9.4, SAS Institute. Inc., Cary, NC). For growth performance measurements, pen was used as the experimental unit and weight block was the random effect. The statistical model included block, type of oil, supplementation and the interaction between oil type and supplementation. For serum measurements, individual pig was considered the experimental unit and the data were analyzed using the Proc MIXED Procedure. The statistical model included block, type of oil, supplementation, day of collection (as repeated measure) and the appropriate interactions. Preplanned comparisons of individual treatments were conducted using the least significant difference method following a significant *F*-test. Statistical significances were considered at *P* < 0.05 and tendencies at 0.05 ≤ *P* ≤ 0.10.

## Results

### Chemical analysis of peroxidized soybean oil

Results of chemical analysis of control and peroxidized soybean oil showed marked differences in peroxidation measures (Table 2). Initial peroxide value for the peroxidized oil was higher than the control oil (141.6 vs. 4.1 mEq/kg fat). Likewise, peroxide values after exposure of oil to air and heat for 4 and 20 h (AOM) in peroxidized oil were increased relative to control oil (3.6 vs. 158 and 4.6 vs. 41.1 for 4 and 20 h, respectively). Anisidine value, hexanal, and 2,4-decadienal levels were highly increased in the peroxidized oil compared to control oil (1.7 vs. 106, < 5 vs. 99 mg/kg and 8 vs. 720 mg/kg, respectively).

**Table 2:** Analyzed composition of experimental soybean oil sources^a^

Item	Control	Peroxidized
Moisture, %	0.10	0.11
Insoluble impurities, %	0.06	none
Unsaponifiable matter, %	0.38	0.34
Free fatty acids, %	0.02	0.09
Peroxide value, mEq/kg fat		
Initial^b^	4.1	141.6
4 h AOM^c^	3.6	158
20 h AOM	4.6	41.2
Anisidine value^d^	1.7	106
Oxidative stability index (OSI), h^e^	37	2.4
Hexanal, mg/kg	< 5	99
2, 4-decadienal, mg/kg	8	720

^a^Analysis of oxidative stability index, hexanal, and 2,4-decadienal were conducted by Kemin Industries (Des Moines, IA), while the other analyses were conducted by New Jersey Feed Laboratory, Inc. (Trenton, NJ)

^b^The New Jersey Feed Laboratory, Inc. (method 965.33 [34]) and Kemin Industries, Inc. both analyzed initial peroxide value and the mean of these values is reported here

^c^Active oxygen method, Cd 12–57 [35])

^d^Anisidine value is a relative measure used to determine aldehyde content of peroxidized oils

^e^OSI, method Cd 12B-92 [35]

### Growth performance

Two pigs, one from a pen fed the peroxidized oil treatment plus vitamin E, and one from a pen fed the control oil diet plus polyphenols, were removed from the study due to failure to thrive and losses in body weight (BW). No interactions between lipid peroxidation and supplementation were observed for growth performance (*P* > 0.05). Peroxidation and antioxidant supplementation (*P* ≥ 0.05) did not impact BW on day 7 or 14 (Table 3). However, lipid peroxidation reduced BW (*P* < 0.001) on day 21 (− 7.8%), 28 (− 13.8%) and 35 (− 19.1%). Average daily gain (ADG) was reduced (*P* < 0.05) by lipid peroxidation during week 2 (− 7.6%), 3 (− 27.3%), 4 (− 32.1%), 5 (− 35.3%), phase 2 (− 32.4%) and overall (− 26.9%). In addition, ADG tended to be increased by supplementation of antioxidants (*P* = 0.063). Average daily feed intake (ADFI) tended to be reduced by lipid peroxidation for week 2 (*P* = 0.093; − 5.6%), and was reduced for week 3 (*P* < 0.001; − 13.1%), 4 (*P* < 0.001; − 22.8%), 5 (*P* < 0.001; − 23.1%), phase 2 (*P* < 0.001; − 22.2%), and overall (*P* < 0.001; − 18.7%). Lipid peroxidation reduced (*P* < 0.05) G:F for week 3 (− 15.5%), 4 (− 12.4%), 5 (− 15.9%), phase 2 (− 13.5%) and overall (− 10.1%). In addition, G:F was reduced (− 10%) by vitamin E supplementation for week 3 (*P* = 0.019) and tended to be improved (+ 3%) by polyphenol supplementation for the overall period (*P* = 0.074). During the first week of the study, lipid peroxidation reduced (− 8.5%) G:F in pigs fed vitamin E and increased G:F (+ 14%) in pigs fed the polyphenol treatment (interaction, *P* = 0.084).

**Table 3:** Growth performance of piglets fed control oil or peroxidized oil supplemented without or with antioxidants^1^

Item	Dietary treatments									
	Control oil			Peroxidized oil			SEM	*P*-value^2^		
	CON	VITE	POL	CON	VITE	POL		O	S	O ×S
Body weight, kg										
Day 0	6.62	6.63	6.61	6.61	6.62	6.62	0.390	0.880	0.600	0.792
Day 7	7.63	7.44	7.40	7.52	7.38	7.57	0.390	0.982	0.128	0.189
Day 14	10.23	10.06	10.24	9.81	9.82	10.25	0.410	0.129	0.193	0.445
Day 21	13.4	13.0	13.2	12.0	11.8	12.7	0.478	< 0.001	0.110	0.254
Day 28	17.6	17.3	17.5	14.8	14.6	15.8	0.613	< 0.001	0.272	0.414
Day 35	23.3	23.0	23.2	18.4	18.3	19.5	0.736	< 0.001	0.356	0.489
Average daily gain, kg/d										
Day 0 to 7	0.145	0.117	0.112	0.130	0.108	0.136	0.013	0.975	0.107	0.218
Day 7 to 14	0.372	0.374	0.396	0.327	0.344	0.383	0.020	0.045	0.063	0.654
Day 0 to 14 (Phase 1)	0.258	0.245	0.258	0.229	0.228	0.260	0.015	0.133	0.180	0.442
Day 14 to 21	0.450	0.413	0.423	0.311	0.279	0.347	0.031	< 0.001	0.219	0.364
Day 21 to 28	0.602	0.615	0.613	0.400	0.399	0.443	0.036	< 0.001	0.732	0.806
Day 28 to 35	0.814	0.814	0.821	0.509	0.538	0.537	0.031	< 0.001	0.788	0.851
Day 14 to 35 (Phase 2)	0.622	0.614	0.619	0.407	0.405	0.442	0.023	< 0.001	0.586	0.625
Day 0 to 35 (Overall)	0.477	0.467	0.475	0.336	0.334	0.369	0.018	< 0.001	0.357	0.492
Average daily feed intake, kg/d										
Day 0 to 7	0.194	0.167	0.167	0.182	0.167	0.177	0.012	0.930	0.171	0.626
Day 7 to 14	0.447	0.437	0.461	0.413	0.416	0.440	0.019	0.093	0.347	0.914
Day 0 to 14 (Phase 1)	0.321	0.302	0.317	0.297	0.294	0.308	0.014	0.219	0.511	0.798
Day 14 to 21	0.786	0.785	0.764	0.657	0.695	0.676	0.036	< 0.001	0.810	0.798
Day 21 to 28	0.851	0.861	0.832	0.619	0.654	0.692	0.037	< 0.001	0.746	0.454
Day 28 to 35	1.194	1.165	1.135	0.884	0.903	0.901	0.039	< 0.001	0.804	0.526
Day 14 to 35 (Phase 2)	0.906	0.888	0.872	0.671	0.695	0.709	0.031	< 0.001	0.995	0.426
Day 0 to 35 (Overall)	0.672	0.653	0.650	0.521	0.535	0.549	0.022	< 0.001	0.966	0.450
Gain:feed, kg/kg										
Day 0 to 7	0.746	0.692	0.666	0.709	0.633	0.759	0.042	0.963	0.181	0.084
Day 7 to 14	0.833	0.852	0.852	0.788	0.829	0.872	0.028	0.379	0.112	0.547
Day 0 to 14 (Phase 1)	0.806	0.809	0.812	0.764	0.777	0.842	0.027	0.427	0.151	0.241
Day 14 to 21	0.568	0.523	0.553	0.469	0.406	0.513	0.030	< 0.001	0.019	0.250
Day 21 to 28	0.710	0.713	0.735	0.642	0.605	0.643	0.030	0.001	0.599	0.798
Day 28 to 35	0.686	0.699	0.726	0.577	0.597	0.601	0.029	< 0.001	0.520	0.915
Day 14 to 35 (Phase 2)	0.688	0.693	0.712	0.605	0.580	0.627	0.021	< 0.001	0.241	0.705
Day 0 to 35 (Overall)	0.710	0.714	0.732	0.641	0.624	0.675	0.018	< 0.001	0.074	0.556

^1^Values represent least squares means of 8 pens with 4 pigs (2 gilts and 2 barrows) per pen. Antioxidant supplementation consisted of control (CON), vitamin E (VITE) or polyphenols (POL)

^2^Effects abbreviations: *O* oil, *S* supplementation, *O × S* oil × supplementation

### Serum oxidative status markers

Lipid peroxidation, supplementation and day of sampling did not significantly affect serum 8-OHdG (*P* ≥ 0.05; Table 4). Serum protein carbonyl was increased (*P* < 0.001) on day 35 when peroxidized oil was fed (3.35 vs. 2.41 pmol/mg protein for peroxidized and control oil, respectively), but peroxidation of oil did not affect protein carbonyl when measured on day 14 (2.08 vs. 1.98 pmol/mg protein, for peroxidized and control oil, respectively). Feeding peroxidized oil decreased serum MDA concentration on day 14 (8.43 vs. 12.7 8 μmol/L for peroxidized and control oil, respectively), but did not impact MDA concentration when measured on day 35 (12.5 vs. 12.0 μmol/L) (interaction; *P* < 0.001).

**Table 4:** Serum oxidative stress markers in piglets fed control or peroxidized oil supplemented without or with antioxidants^1^

Item	Treatments													
	Control oil			Peroxidized oil			SEM	*P-*value^2^						
	CON	VITE	POL	CON	VITE	POL		O	D	S	O × D	O × S	S × D	O × S × D
8-hydroxydeoxyguanosine, ng/mL														
Day 14	5.1	5.5	5.1	4.6	5.1	4.2	0.79	0.128	0.653	0.705	0.458	0.298	0.513	0.856
Day 35	4.8	4.9	5.7	4.8	5.3	4.8								
Protein carbonyl, pmol/mg protein														
Day 14	2.09	1.91	1.93	1.89	2.24	2.10	0.22	< 0.001	< 0.001	0.446	< 0.001	0.231	0.553	0.099
Day 35	2.28	2.58	2.37	3.25	3.11	3.69								
Malondialdehyde, μmol/L														
Day 14	11.7	13.9	12.5	8.6	8.3	8.4	0.89	< 0.001	0.004	0.165	0.001	0.132	0.457	0.460
Day 35	11.2	12.2	14.1	11.9	12.0	12.1								
Total antioxidant capacity, Cu reducing equivalents/mL														
Day 14	288	323	303	295	255	261	15.87	< 0.001	< 0.001	0.538	0.924	0.045	0.025	0.305
Day 35	335	355	379	317	310	335								

^1^Values represent least squares means of 8 pigs. Antioxidant supplementation consisted of control (CON), vitamin E (VITE) or polyphenols (POL)

^2^Effects abbreviations: *O* oil source, *D* day, *S* supplementation, and their interactions

### Serum vitamin E concentrations and total antioxidant capacity

**Fig. 1:**
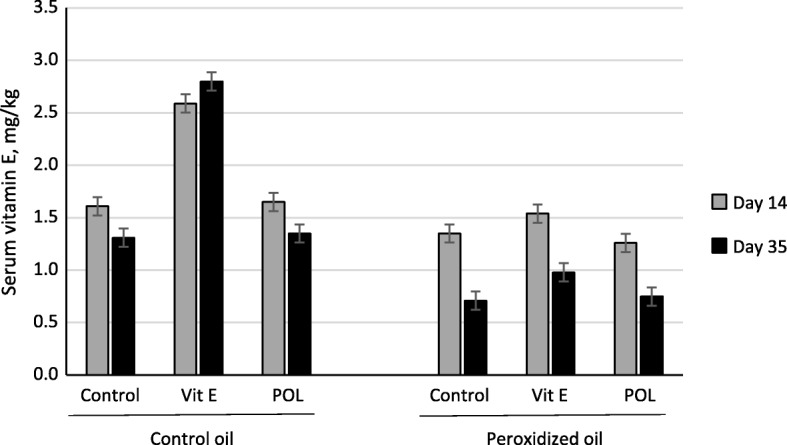
Serum vitamin E concentrations in piglets fed dietary control oil or peroxidized oil supplemented without (Control) or with vitamin E (Vit E) or polyphenols (POL). Each bar represents least squares means of 8 pigs. Significant effects were observed for the oil source by supplementation interaction (*P* < 0.001), the supplementation ×day interaction (*P* < 0.001) and the individual main effects of oil source, supplementation, and day of measurement (*P* < 0.001)

Lipid peroxidation decreased serum vitamin E concentrations by 41.8% (*P* < 0.001; Fig. 1) and this reduction in vitamin E concentration was greater on day 35 compared to day 14 (day ×peroxidation interaction, *P* < 0.001). Supplementation of vitamin E increased serum vitamin E concentrations (*P* < 0.001) compared to control and polyphenols treatments (1.98 vs. 1.25 and 1.26 mg/kg, respectively) and this increase was greater for the control diet (84.6%) compared to the diets with peroxidized oil (22.3%) (interaction, *P* < 0.001). The three-factor interaction between lipid peroxidation, supplementation and day of measurement was not significant (*P* = 0.101).

Serum TAC was decreased by 10.6% (*P* < 0.001) by lipid peroxidation compared to control oil (Table 4). Additionally, serum TAC was greater when measured on day 35 compared to day 14 (+ 18%; *P* < 0.001). Supplementation of vitamin E (*P* = 0.046) and polyphenols (*P* = 0.065) to diets containing control soybean oil increased TAC, but this was not the case when supplemented to diets containing peroxidized oil (interaction, *P* = 0.045). In addition, TAC was increased by polyphenol supplementation compared to the control diet (*P* = 0.016) when measured on day 35, but not day 14 (interaction, *P* = 0.025).

### Serum cytokines

Lipid peroxidation reduced the serum concentrations of pro-inflammatory cytokines (IL-1α, IL-1β, IL-2, IL-6, IL-12, and IL-18) and anti-inflammatory cytokines (IL-1ra, IL-4, and IL-10) on day 14, but they were increased by lipid peroxidation on day 35 (interaction, *P* < 0.05; Table 5). Interleukin-1*α*, IL-1*β*, IL-1ra, IL-2, IL-4, IL-6, IL-10, IL-12, and IL-18 were reduced and IFN-*γ* was increased on day 35 compared to day 14 (*P* < 0.05). Dietary vitamin E and polyphenols tended to reduce (*P* < 0.10) IL-1*β*, IL-1ra, IL-2 and IL-4 when compared with the control treatment.

**Table 5:** Serum immune status of piglets fed control or peroxidized oil supplemented without or with antioxidants^1^

Item	Treatments											
	Control oil			Peroxidized oil			SEM	*P*-value^2^				
	CON	VITE	POL	CON	VITE	POL		O	D	S	O × D	O × S × D
Interferon-*γ*, pg/mL												
Day 14	14,510	4936	15,596	10,765	6032	7193	3330	0.408	0.034	0.116	0.158	0.337
Day 35	14,757	7987	14,824	11,460	10,368	14,351						
Interleukin-1*α*, pg/mL												
Day 14	132	96	97	73	56	63	32	0.601	0.017	0.351	0.009	0.703
Day 35	47	36	32	84	77	4						
Interleukin-1*β*, pg/mL												
Day 14	2030	1139	891	612	415	518	350	0.307	0.052	0.043	0.003	0.230
Day 35	388	337	247	735	1092	394						
Interleukin-1ra, pg/mL												
Day 14	1254	861	737	637	615	674	160	0.939	0.022	0.083	0.002	0.239
Day 35	507	414	369	932	635	607						
Interleukin-2, pg/mL												
Day 14	1412	406	739	529	377	489	203	0.481	0.010	0.052	0.015	0.149
Day 35	244	235	203	642	438	259						
Interleukin-4, pg/mL												
Day 14	8055	2040	5686	3931	2307	2403	1343	0.774	0.018	0.056	0.010	0.357
Day 35	1391	1162	901	4510	2956	1747						
Interleukin-6, pg/mL												
Day 14	254	154	155	100	73	85	46	0.361	0.006	0.420	0.005	0.590
Day 35	43	34	30	85	140	37						
Interleukin-8, pg/mL												
Day 14	623	757	985	427	507	673	180	0.135	0.827	0.801	0.422	0.799
Day 35	672	821	730	755	607	491						
Interleukin-10, pg/mL												
Day 14	3840	2519	2450	1634	1124	1451	657	0.142	0.001	0.317	0.020	0.544
Day 35	782	566	623	1464	1063	495						
Interleukin-12, pg/mL												
Day 14	1588	1310	1193	996	1044	998	147	0.198	0.004	0.263	0.008	0.092
Day 35	803	817	958	1232	877	873						
Interleukin-18, pg/mL												
Day 14	3384	1617	2450	1871	1352	1642	542	0.764	0.021	0.118	0.021	0.459
Day 35	1104	963	787	2265	1585	1005						
Tumor necrosis factor-*α*, pg/mL												
Day 14	23	251	91	199	35	16	59	0.428	0.419	0.885	0.794	0.023
Day 35	89	42	131	59	69	70						

^1^Values analyzed represent least squares means of 8 pigs. Antioxidant supplementation consisted of control (CON), vitamin E (VITE) or polyphenols (POL)

^2^Effects abbreviations: *O* oil source, *D* day, *S* supplementation, and their interactions; *O × S* oil × supplementation (*P* ≥ 0.05) and S × D = supplementation × day (*P* ≥ 0.05)

## Discussion

Feeding peroxidized oil to pigs negatively affects growth performance [12, 36, 37] and this decrease appears to be directly related to the extent of peroxidation. In the present study, growth performance was compromised to the largest extent from day 14 through day 35 (Phase 2), but effects were limited for the initial 14 days (Phase 1). The first phase diet was complex in composition, containing whey permeate as a source of lactose, spray-dried plasma protein, and other highly digestible animal-based proteins ingredients. These ingredients are highly palatable, which is especially important for the first 2 weeks after weaning [38, 39]. Perhaps these ingredients allowed pigs to maintain feed intake in the presence of negative odor and taste associated with aldehydes in peroxidized oil that are responsible for rancid odor and off flavor [40]. On the other hand, there may be a threshold beyond which the impact of peroxidized lipids manifests itself. The fact that detrimental effects of dietary peroxidized soybean oil was expressed more in the long term is supported by Anjum et al. [14] and Lu et al. [30] in broilers and pigs, respectively. DeRouchey et al. [36], Liu et al. [11] and Rosero et al. [12] reported reductions in ADFI in pigs when feeding peroxidized lipids and these effects were dose-dependent. The reduction in growth rate is due, in part, to reduced feed intake, but is also likely associated with the negative impacts in the intestine caused by dietary peroxidized oil when fed to piglets [12]. In addition, peroxidized lipids have been shown to decrease digestibility of fat and gross energy in pigs [13], which can negatively impact daily gain and feed efficiency. The degree of lipid peroxidation in the present study was consistent with previous reports and is of practical relevance to the animal production industry [41].

The addition of peroxidized oil in the diet for pigs causes oxidative stress [9, 10], but the supplementation of dietary vitamin E [42] and polyphenols [43] could ameliorate the negative effects caused by oxidation [9, 37]. In the present study, supplementation of vitamin E was not effective in improving pig performance, regardless of the peroxidation status of the soybean oil fed. Feeding polyphenols tended to improve gain:feed in pigs fed peroxidized lipids during the first week of the study only and tended to improve gain:feed for the overall study, regardless of the oil source. These results agree partially with those published by Boler et al. [9], who found no effects of supplementation with tert-butylhydroquinone and ethoxyquin blend antioxidant to a diet containing peroxidized corn oil in finishing pigs. Rooke et al. [44] reported that an antioxidant blend or antioxidant blend plus vitamin E improved growth performance when feeding 5% of dietary peroxidized oil and showed regeneration of vitamin E by vitamin C and glutathione peroxidase. Peroxidation products could reduce the function of fat-soluble vitamins [30].

Several authors reported that peroxidized oil reduced serum concentrations of vitamin E due to catabolism of the vitamin E during oxidative stress [8, 9, 17, 37, 45]. This fact agrees with the results found in the present study. Serum vitamin E was greatly reduced with peroxidation, especially after prolonged exposure, showing the ability of vitamin E to react against oxidation. In addition, the reduction in feed intake in pigs fed peroxidized oil (and thus reduced intake of vitamin E) contributes to decreased serum vitamin E concentrations. Soybean oil contains significant amounts of tocopherols, which are destroyed by peroxidation. Lindblom and coworkers [13] reported a 48% reduction of total tocopherols in thermally peroxidized soybean oil, which will also contribute to reduced serum vitamin E concentrations. Similarly, Yen et al. [16] reported lower serum vitamin E concentrations in rats consuming a diet with 15% of deep-frying oils. Vitamin E is a part of all antioxidant actions present in plasma, body fluids, and cell membranes. Supplementation of vitamin E increased serum vitamin E concentrations in both control pigs and pigs fed peroxidized lipids, but this increase was much smaller in the latter. On the other hand, supplementation of polyphenols did not impact serum vitamin E concentration, regardless of the peroxidation status of the oil fed, suggesting that polyphenols were not able to regenerate vitamin E once it was oxidized. This could be related to low absorption rate of dietary polyphenols as reported by Gessner et al. [46], the type of polyphenols used, or functioning of polyphenols as an antioxidant that is independent from vitamin E.

Total antioxidant activity broadly measures antioxidant actions in the body including hydrophilic and lipophilic antioxidants and can be highly dependent on uric acid concentrations. On the other hand, TAC does not measure antioxidant enzymes, such as superoxide dismutase, glutathione peroxidase, and catalases [47], which are important components of the antioxidant system. Vitamin E is positively correlated with TAC and partially contributed to TAC results in the present study. Indeed, TAC was reduced by peroxidation, similar to serum vitamin E concentrations. Values of TAC were higher on day 35 compared to day 14, indicating increased antioxidant capacity as pigs grew and reflecting increased feed consumption. In addition, serum TAC was increased when vitamin E and polyphenols were supplemented to pigs fed control oil demonstrating effectiveness of both in improving antioxidant status. However, serum TAC was not impacted when vitamin E or polyphenols were supplemented to pigs fed peroxidized oil, suggesting that antioxidants effectively reacted against oxidation. Interestingly, on day 35, serum concentrations of TAC were higher for the polyphenols treatment than the control or dietary vitamin E treatment, suggesting that antioxidant efficiency of polyphenols increased with time. Contrarily, Lipiński et al. [48] concluded that polyphenols are deposited in the organs which participate in their metabolism, with plasma concentrations remaining lower during long periods or high doses in pigs. Moreover, no improvements in TAC were reported by Gessner et al. [46], who used 1% of dietary polyphenols in diets of piglets. Also, during metabolism of polyphenols (flavonoids) uric acid is produced as part of TAC [47]. Thus, high levels of TAC resulting from polyphenols treatments may be due to metabolic products derived from the commercial polyphenols used in this study.

Peroxidized lipids damage proteins, leading to malfunction and alteration in their structure, increasing protein carbonyls. Protein carbonyl is a biomarker with high stability, and it is formed during the early period of oxidative stress [49]. In the present study, protein carbonyl in serum was increased by lipid peroxidation, particularly on day 35, but supplementation with antioxidants did not ameliorate this effect. Likewise, Lindblom et al. [21] and Lu et al. [30] reported increased protein carbonyl in serum of pigs fed peroxidized oil. Boler et al. [9] did not observe differences in protein carbonyls in plasma when using a synthetic antioxidant blend supplemented to diets containing peroxidized corn oil, when compared with a peroxidized corn oil diet without antioxidant in pigs. A study conducted by Lu et al. [30] showed significant decreases in plasma carbonyls on day 55 in growing pigs fed peroxidized lipids when using dietary vitamin E in combination with a synthetic antioxidant blend, but this was not the case on day 118, indicating that long-term supplementation of peroxidized oil may overwhelm the antioxidant system resulting in increased markers of oxidative stress.

Malondialdehyde is a three-carbon cytotoxic molecule, produced by peroxidation of polyunsaturated fatty acids [49]. The serum concentrations of MDA, as a marker of lipid peroxidation status, have been shown to increase due to the consumption of peroxidized oil as reported in previous studies [9, 14, 16, 30]. In the present study, serum MDA concentrations decreased with feeding with peroxidized oil when measured on day 14 and 35, and MDA levels were higher on day 35 than day 14. In contrast, Chang et al. [17] did not observe differences in serum MDA due to lipid peroxidation in weaned piglets. Hung et al. [37] conducted a meta-analysis using 23 studies conducted in swine and reported that serum MDA concentrations generally increased with the supplementation of peroxidized lipids. However, only few of those studies reported serum MDA concentrations as an outcome variable in that meta-analysis.

8-OHdG is an oxidized nucleoside of DNA detected during DNA oxidation [49]. Comparing with other oxidative status markers tested in this study, serum 8-OHdG was not affected by lipid peroxidation or antioxidant supplementation. Likewise, Lindblom et al. [21] and Chang et al. [17] did not report differences in DNA damage using various levels of peroxidation of supplemental oil. The site of DNA damage occurs in nuclear and mitochondrial DNA in tissue and in DNA of lymphocytes [50, 51]. Lymphokines which are derived from lymphocyte stimulation and synthesized by lymphocytes clones, include IL-1, IL2, IL-3, IL-4, IL-5, IL-6, TNF-*α* and IFN-*γ* [52]. In addition, peroxidation caused lipid oxidation and inflammation in intestinal cells due to activation of nuclear factor kappa B, producing an increase in cytokines levels [53]. In the present study, cytokines increased with lipid peroxidation only on day 35 and could be related to the reduction in growth performance observed during the end of the study. This suggests that peroxidation over a prolonged period may have induced inflammation in pigs, with a subsequent negative impact on growth performance. It should be noted that serum measurements are single point measurements in time, whereas growth performance is cumulative and provides an assessment of total impacts over time. Similar to our results, Rosero et al. [12] did not find differences in the pro-inflammatory cytokine TNF-α when peroxidized oils were added in the diet for piglets. The high levels of the majority of cytokines on day 14 compared to day 35 may be due to the early weaning effects on pigs [54]. Interestingly, cytokines increased with peroxidation on day 35 and could be related to the reduction in growth performance at the end of the study.

## Conclusion

The addition of peroxidized soybean oil negatively affected growth performance, especially during the later stages of the study. Moreover, feeding peroxidized lipids reduced serum vitamin E concentrations and total antioxidant capacity, increased protein oxidation, and increased serum cytokine concentrations after 35 days of exposure. Supplementation of vitamin E increased serum concentrations of vitamin E and both vitamin E and polyphenols improved total antioxidant capacity, especially in pigs fed control diets. Nonetheless, no significant improvements in growth performance or oxidative status were detected at the concentrations of vitamin E and polyphenols supplemented.

## Data Availability

The dataset analyzed in the present study can be made available from the corresponding author upon reasonable request.

## Abbreviations

8-OHdG: 8-hydroxydeoxyguanosine
ADFI: Average daily feed intake
ADG: Average daily gain
AOM: Active Oxygen Method
BCA: Bicinchoninic acid
BW: Body weight
CRE: Copper Reducing Equivalents
DNA: Deoxyribonucleic acid
G:F: Gain to feed ratio
IFN: Interferon
IL: Interleukin
MDA: Malondialdehyde
NRC 2012: Nutrient Requirements of Swine 2012
TAC: Total antioxidant capacity
TBHQ: Tertiary butyl hydroquinone
